# Enrofloxacin Shifts Intestinal Microbiota and Metabolic Profiling and Hinders Recovery from Salmonella enterica subsp. *enterica* Serovar Typhimurium Infection in Neonatal Chickens

**DOI:** 10.1128/mSphere.00725-20

**Published:** 2020-09-09

**Authors:** Boheng Ma, Xueran Mei, Changwei Lei, Cui Li, Yufeng Gao, Linghan Kong, Xiwen Zhai, Hongning Wang

**Affiliations:** a College of Life Sciences, Sichuan University, Chengdu, People’s Republic of China; b Key Laboratory of Bio-Resource and Eco-Environment of Ministry of Education, Chengdu, People’s Republic of China; c Animal Disease Prevention and Food Safety Key Laboratory of Sichuan Province, Chengdu, People’s Republic of China; d Sichuan Water Conservancy Vocational College, Chengdu, People’s Republic of China; Antimicrobial Development Specialists, LLC

**Keywords:** gut microbiota, enrofloxacin, metabolome, chicken, *Salmonella*

## Abstract

Enrofloxacin is an important antibiotic used for prevention and treatment of *Salmonella* infection in poultry in many countries. However, oral administration of enrofloxacin may lead to the alterations in the microbiota and metabolome in the chicken intestine, thereby reducing colonization resistance to the *Salmonella* infection. To study the effect of enrofloxacin on *Salmonella* in the chicken cecum, we used different concentrations of enrofloxacin to feed 1-day-old chickens, followed by oral challenge with Salmonella enterica subsp. *enterica* serovar Typhimurium (*S.* Typhimurium). We then explored the distribution pattern of *S.* Typhimurium in cecum contents *in vivo* and analyzed the microbial community structure of cecum contents using microbial 16S amplicon sequencing. Untargeted metabolomics was used to explore the gut metabolome on day 14. *Faecalibacterium* and *Anaerostipes*, which are closely related to the chicken intestinal metabolome, were screened using a multi-omics technique. The abundance of *S.* Typhimurium was significantly higher in the enrofloxacin-treated group than in the untreated group, and *S.* Typhimurium persisted longer. Moreover, the cecal colony structures of the three groups exhibited different characteristics, with *Lactobacillus* reaching its highest abundance on day 21. Notably, *S.* Typhimurium infection is known to affect the fecal metabolome of chickens differently. Thus, our results suggested that enrofloxacin and *Salmonella* infections completely altered the intestinal microbiota and metabolism of chickens.

**IMPORTANCE** In this study, we examined the effects of *S.* Typhimurium infection and enrofloxacin treatment on the microbiota and metabolite synthesis in chicken cecum, in order to identify target metabolites that may promote *S.* Typhimurium colonization and aggravate inflammation and to evaluate the important microbiota that may be associated with these metabolites. Our findings may facilitate the use of antibiotics to prevent *S.* Typhimurium infection.

## Abstract (precis)

In this study, we examined the effects of *S.* Typhimurium infection and enrofloxacin treatment on the microbiota and metabolite synthesis in chicken cecum, in order to identify target metabolites that may promote *S.* Typhimurium colonization and aggravate inflammation and to evaluate the important microbiota that may be associated with these metabolites. Our findings may facilitate the use of antibiotics to prevent *S.* Typhimurium infection.

## INTRODUCTION

Foodborne diseases are associated with high morbidity and mortality rates worldwide and pose major challenges to food safety and economics (1). *Salmonella* is an important foodborne pathogen of the family *Enterobacteriaceae*, which causes millions of infections worldwide each year, both in humans and in livestock. *Salmonella* has been shown to cause intestinal inflammation and barrier dysfunction in chickens, thereby affecting chicken performance (2). Currently, more than 2,600 serotypes of *Salmonella* are known, with varying pathogenicity and host specificity. Salmonella enterica subsp. *enterica* serovar Typhimurium (*S.* Typhimurium) is a typical representative of non-host-specific *Salmonella* found in poultry. The main route of infection with *Salmonella* in poultry is the fecal-oral route. For multiserotype infections, bacteria mainly colonize in the ceca of poultry. After colonizing in the intestines, these bacteria invade the intestinal epithelial cells and dendritic cells and reach the submucosa to be phagocytosed by macrophages.

Owing to the widespread use of antibiotics in agriculture and animal husbandry, *Salmonella* resistance has rapidly increased. In 2013, China alone consumed approximately 927,000 tons of antibiotics (3). Moreover, the consumption of antibiotics as veterinary drugs increased from 46% to 52% between 2007 and 2013. Enrofloxacin, florfenicol, lincomycin, and amoxicillin are among the most commonly consumed veterinary antibiotics in China (4). Enrofloxacin is the main antibiotic used for treating *Salmonella* infection in poultry in many countries, and the residual concentration of enrofloxacin in chicken manure in China was found to be as high as 1,421 mg/kg (5).

Enrofloxacin affect the composition and number of bacterial species in the chicken gut (6), thereby altering resistance to pathogen invasion. Antibiotics reduce the number of butyrate-producing Clostridium perfringens bacteria in the intestinal microbiota, shifting the host cells from oxidative metabolism to lactic acid fermentation and increasing the level of lactic acid in the intestine; *Salmonella* then gains a colonization advantage by using lactic acid (7). Intestinal metabolism is also affected by the gut microbiota composition. Using antibiotics increases fucose and sialic acid contents in the intestine, thereby affecting *Salmonella* and Clostridioides difficile colonization (8, 9). Similarly, antibiotics also decrease the levels of secondary bile acids in the intestine and inhibit C. difficile colonization (9).

Indeed, bacteria residing in the gut can mediate the colonization of *S.* Typhimurium in the body by producing short-chain fatty acids (SCFAs), such as propionic acid, which inhibits *S.* Typhimurium growth by influencing the intracellular pH (10). By producing SCFAs, *Lactobacillus* inhibits *Salmonella* colonization in the colon and jejunum, accelerates *Salmonella* clearance from stools, and reduces *Salmonella* transfer from the small intestine to the spleen (11). The concentrations of SCFAs are inversely proportional to the degree of inflammation (12). Some *Enterobacteriaceae* in the normal gut microbiota can mediate *Salmonella* multiplication through competition for oxygen (13).

Accordingly, in this study, we investigated the effects of enrofloxacin on *Salmonella* colonization in chickens and the relationship between intestinal microbiota and *Salmonella* colonization. We also evaluated changes in microbiota and metabolites in the cecum of enrofloxacin-treated chickens infected with *Salmonella* and used multi-omics association analyses to determine the correlations between microbial community structure and microbial metabolites.

## RESULTS

### Response of *Salmonella* to enrofloxacin.

**FIG 1:**
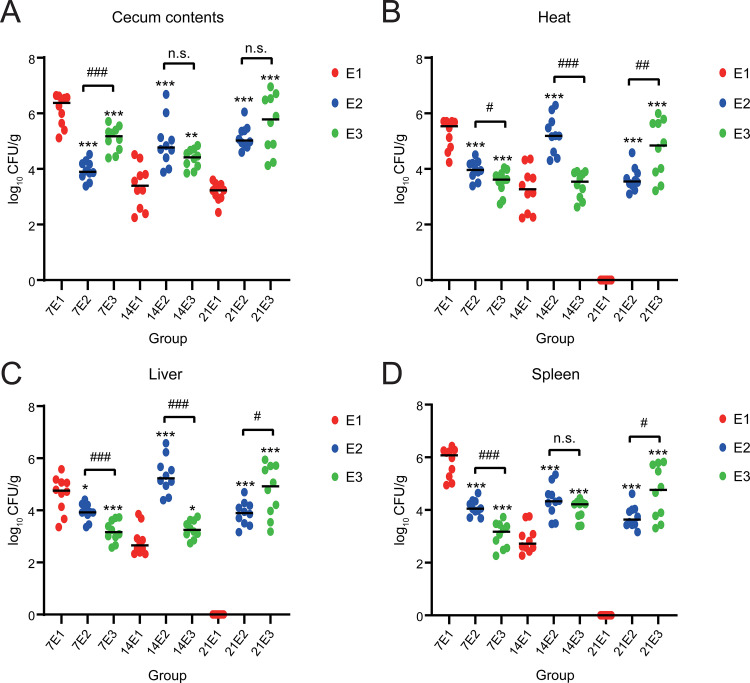
*Salmonella* abundance. Cecum contents (A), heart (B), liver (C), and spleen (D). The *y* axis represents log_10_ copies/g, and the *x* axis represents different groupings. Compared with the same sampling point in group E1, *P* < 0.001, indicated by ***; 0.001 ≤ *P* ≤ 0.01, indicated by **; and 0.01 < *P* ≤ 0.05, indicated by *. *P* > 0.05, no mark. Comparing groups E2 and E3, *P* < 0.001, indicated by ###; 0.001 ≤ *P* ≤ 0.01, indicated by ##; and 0.01 < *P* ≤ 0.05, indicated by #. *P* > 0.05, n.s.

First, *S.* Typhimurium abundance in the samples was evaluated. *S.* Typhimurium was not isolated from C1, C2, or C3 groups. This shows that no environmental *Salmonella* was mixed into samples. On day 7, the highest abundance of *S.* Typhimurium was found in the cecum contents of group E1 (6.128 log_10_ CFU/g), followed by that of groups E3 and E2 (5.090 and 3.945 log_10_ CFU/g, respectively; *P < *0.001). The abundances of *S.* Typhimurium in the heart, liver, and spleen of group E1 were significantly higher than those in the other two groups (*P < *0.001). On day 14, the abundance of *S.* Typhimurium in group E1 (3.371 log_10_ CFU/g) was significantly lower than those in groups E2 (4.829 log_10_ CFU/g) and E3 (4.366 log_10_ CFU/g; *P < *0.01). Moreover, the abundances of *S.* Typhimurium were significantly lower in heart, liver, and spleen samples from group E1 than those from groups E2 and E3, except for heart samples from group E3. On day 21, the relative abundances of *S.* Typhimurium were 3.170, 5.146, and 5.636 log_10_ CFU/g in groups E1, E2, and E3, respectively; that in group E1 was significantly lower than those in the other two groups. However, no *S.* Typhimurium was detected in the heart, liver, or spleen samples of group E1 (Fig. 1).

### Effects of enrofloxacin on the microbial composition and structure.

The 16S rRNA of the cecum colonies was sequenced and analyzed for species diversity of individual samples and differences in diversity between samples. At day 7, the E1 group had the lowest α-diversity while the E3 group had the highest α-diversity. There were significant differences between the groups (*P < *0.05). The microbial community structure of the three groups was clearly separated on the sample clustering tree. One hundred ninety-three operational taxonomic units (OTUs) were found to be common to the three groups, while 22, 24, and 55 OTUs were unique to groups E1, E2, and E3, respectively. On day 14, the α-diversity of the three groups was significantly higher than on day 7 (*P < *0.05). However, the differences in microbial community structure among the three groups gradually decreased. Three hundred ninety-one OTUs were shared among the three experimental groups, whereas 10, 34, and 22 OTUs were unique among the E1, E2, and E3 groups, respectively. At day 21, the E2 group had significantly higher α-diversity than the other two groups (*P < *0.05). The microbial community structures of the three groups were mixed together in the sample clustering tree. Three hundred ninety-six OTUs were common to the three experimental groups, while 15, 44, and 18 OTUs were unique to groups E1, E2, and E3, respectively.

**FIG 2:**
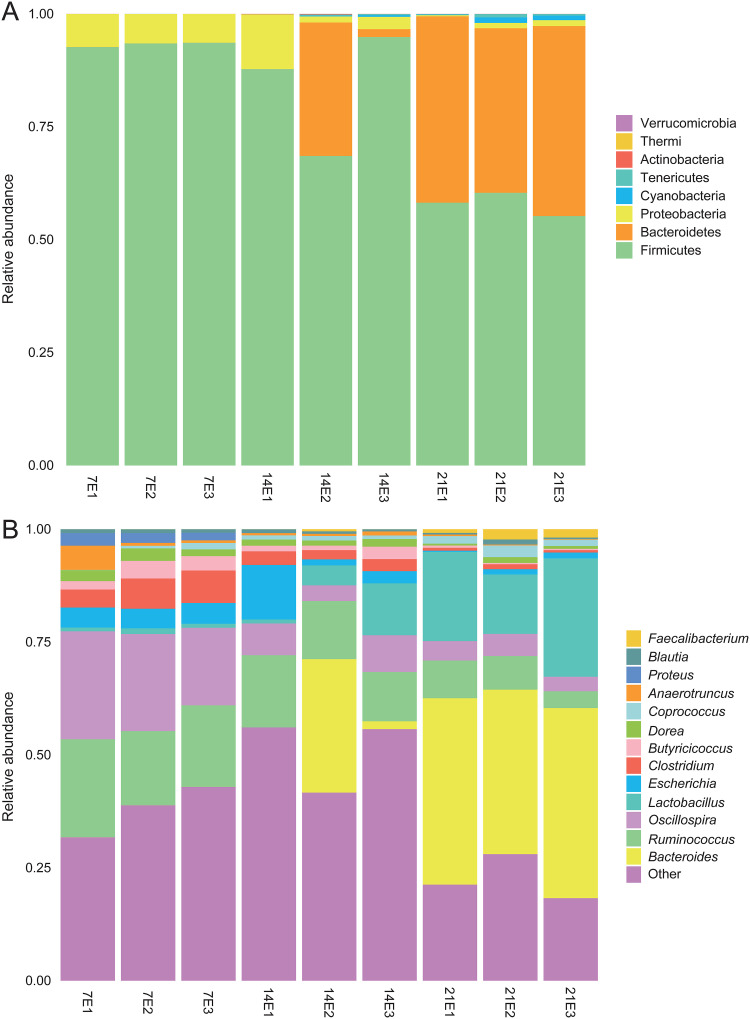
Average relative abundance of microbial species in the cecum at the phylum level (A) and genus level (B).

We also measured the relative abundance of fecal microbiota at the phylum (Fig. 2A) and genus (Fig. 2B) level. At the phylum level, *Firmicutes*, *Bacteroidetes*, and *Proteobacteria* were the three dominant microbiota. Among them, the abundance of *Firmicutes* and *Proteobacteria* decreased with the gradual maturation of the gut microbiota. However, the relative abundance of *Bacteroidetes* gradually increased (see Table S1 in the supplemental material). At the genus level, the relative abundance of *Coprococcus*, *Lactobacillus*, and *Bacteroides* gradually increased over time. In the early stage of the experiment, *Anaerotruncus*, *Oscillospira*, *Ruminococcus*, and *Bacteroides* had higher abundance in the nonantibiotic group than in the antibiotic group. On the 21st day, *Anaerotruncus*, *Butyricicoccus*, and *Ruminococcus* had higher abundance in the nonantibiotic group (Table S2).

10.1128/mSphere.00725-20.2 TABLE S1 Relative abundance of microbial communities in chicken cecum contents at the phylum level. Download Table S1, PDF file, 0.1 MB. Copyright © 2020 Ma et al. 2020 Ma et al. This content is distributed under the terms of the Creative Commons Attribution 4.0 International license.

10.1128/mSphere.00725-20.3 TABLE S2 Relative abundance of microbial communities in chicken cecum contents at the genus level. Download Table S2, PDF file, 0.1 MB. Copyright © 2020 Ma et al. 2020 Ma et al. This content is distributed under the terms of the Creative Commons Attribution 4.0 International license.

**FIG 3:**
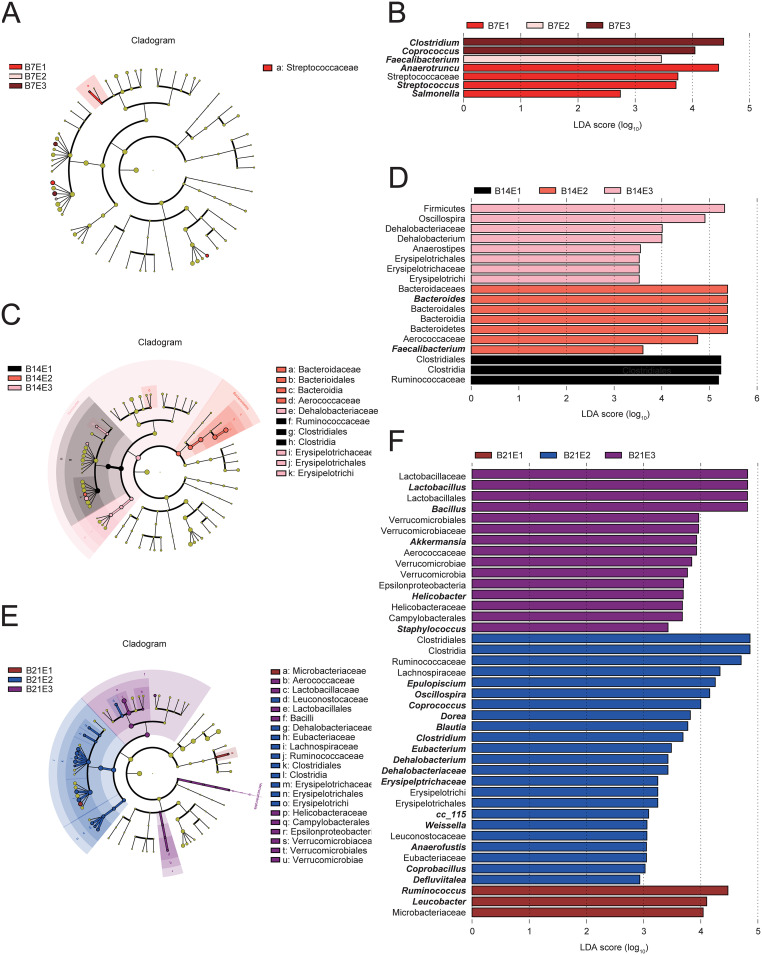
LEfSe analysis of the chicken cecum microbial community in groups E1, E2, and E3 at the three sampling points. LEfSe plots showing microbial strains with significant differences in groups E1, E2, and E3 (A, C, and E). The different groups are represented by different colors, the microbiota that plays an important role in the different groups is represented by nodes of corresponding colors, and the organism markers are indicated by colored circles. The microbiota that does not play a significant role in the different groups is indicated by yellow nodes. From inside to outside, the circles are ordered by species at the level of phylum, class, order, family, and genus. Linear discriminant analysis (LDA) diagram (B, D, and F). The different colors represent microbial groups that play a significant role in groups E1, E2, and E3. Biomarkers with statistical differences are emphasized, with the colors of the histograms representing the respective groups and the lengths representing the LDA score, which is the magnitude of the effects of significantly different species between groups.

Linear discriminant analysis (LDA) effect size (LEfSe) analysis was applied to determine the effect of infection with *S.* Typhimurium following the use of different concentrations of enrofloxacin on the chicken cecum microbiota (LDA] > 2.0). All three experimental groups were heavily enriched with bacteria at the three time points. Interestingly, at day 7, *Salmonella* was an important bacterium that distinguished the E1 group from the other two groups (Fig. 3).

### Metabolomic profiling of the cecum.

**FIG 4:**
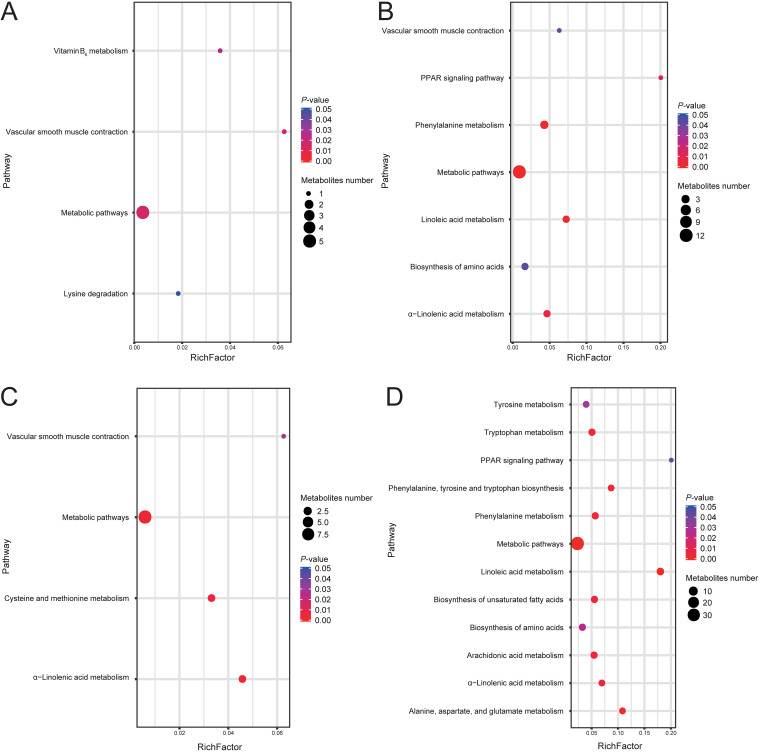

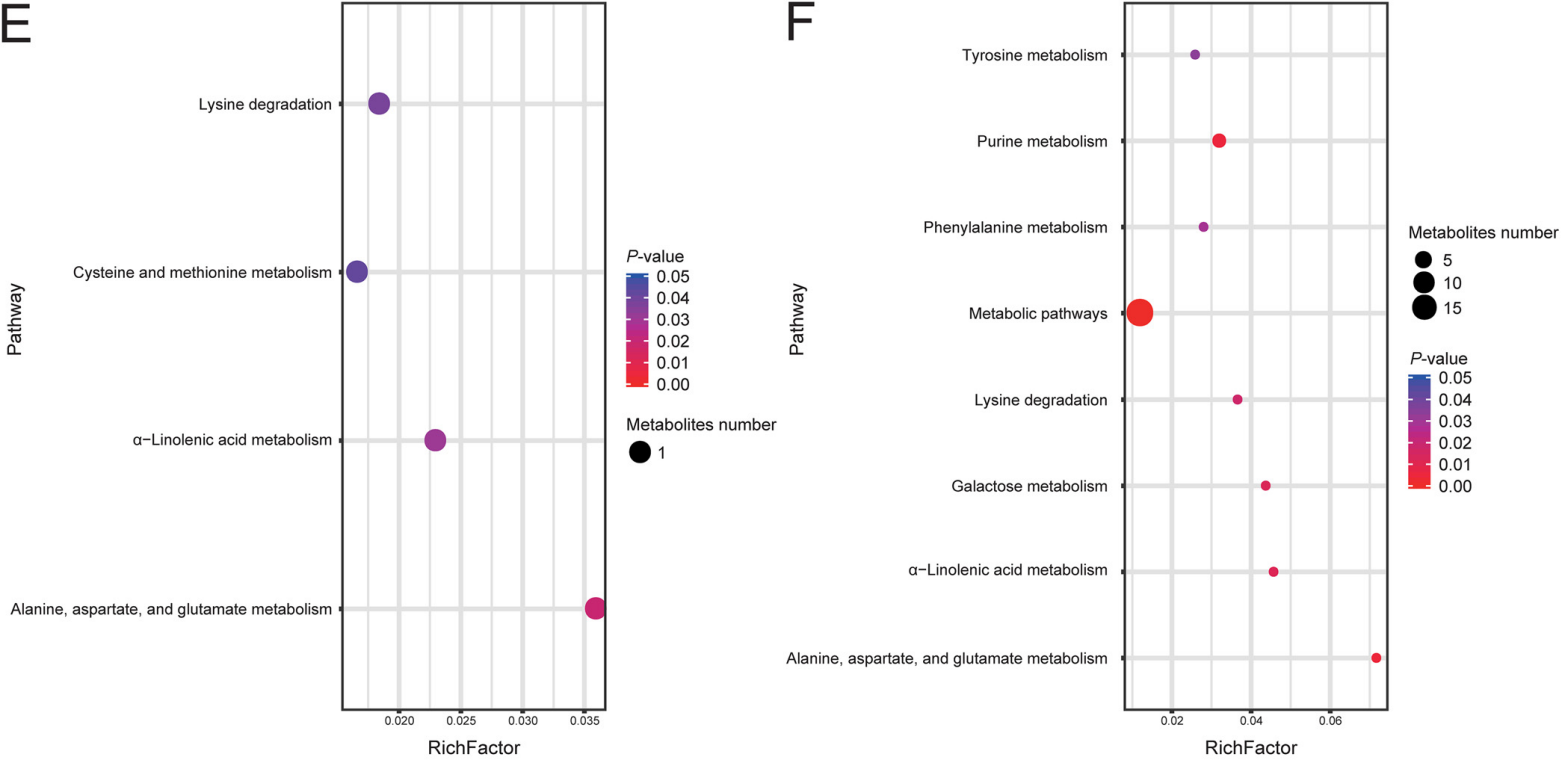
KEGG pathway analysis of metabolites in cecum contents at day 14. The metabolites in the different comparison groups were analyzed for metabolic pathway enrichment on the basis of the KEGG database. The *x* axis (RichFactor) represents the ratio of the number of differential metabolites annotated to the pathway to all the metabolites annotated to the pathway. The size of the dots represents the number of differential metabolites annotated to the pathway. (A) Upregulated metabolites in group E2 versus E1; (B) downregulated metabolites in group E2 versus E1; (C) upregulated metabolites in group E3 versus E1; (D) downregulated metabolites in group E3 versus E1; (E) upregulated metabolites in group E3 versus E2; (F) downregulated metabolites in group E3 versus E2.

We used the nontargeted metabolomic technique to detect metabolites in cecum contents. Partial least-squares discriminant analysis (PLS-DA) results showed significant differences between the metabolites in the different experimental groups (Fig. S1). The number of differential metabolites (variable importance in projection [VIP] ≥1; fold change ≥1.2 or fold change ≤0.83; *q *< 0.05) in each comparison group is shown in Table 1. These differential metabolites were enriched according to the KEGG database. The enrichment results show that the differential metabolites are mainly enriched in short-chain fatty acid metabolism (such as α-linolenic acid metabolism, linoleic acid metabolism, arachidonic acid metabolism, etc.), amino acid metabolism (such as phenylalanine metabolism, tyrosine metabolism, alanine, aspartate, and glutamate metabolism, etc.), the peroxisome proliferator-activated receptor (PPAR) signaling pathway, and unsaturated fatty acids biosynthesis and other metabolic processes (Fig. 4).

**TABLE 1:** Differential metabolite statistics

Analysis mode, comparison group	Significantly different metabolite species (VIP ≥ 1, FC ≥ 1.2, or FC ≤ 0.83, *q* < 0.05)			
	Positive ion mode	Trend	Negative ion mode	Trend
E2 vs E1	486	219↑	271	149↑
		267↓		122↓

E3 vs E1	1,577	605↑	656	252↑
		972↓		404↓

E3 vs E2	554	231↑	328	102↑
		323↓		226↓

10.1128/mSphere.00725-20.1 FIG S1 Partial least-squares discriminant analysis (PLS-DA) fractional scatterplots of the identified metabolites with the first principal component on the horizontal axis and the second principal component on the vertical axis. The number in parentheses indicates the score for that principal component and represents the percentage of the overall variance explained by the corresponding principal component. (A and B) For metabolites in the negative or positive ion mode of group E2 versus E1; (C and D) for metabolites in the negative or positive ion mode of group E3 versus E1; (E and F) for metabolites in the negative or positive ion mode of group E3 versus E2. Download FIG S1, PDF file, 0.3 MB. Copyright © 2020 Ma et al. 2020 Ma et al. This content is distributed under the terms of the Creative Commons Attribution 4.0 International license.

### Correlation analysis of the microbiome and metabolome.

**FIG 5:**
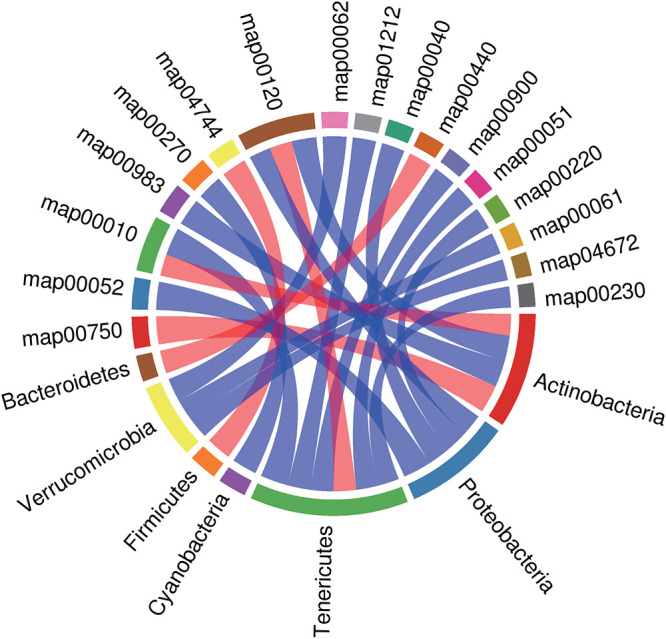
Rank correlation analysis of metabolic pathways and phylum-level microbial groups. The top 20 relationship pairs with the strongest rank correlation (with the largest correlation coefficient absolute value and *P* < 0.05) are shown as chord diagrams. The blue line represents a positive correlation, and the red line represents a negative correlation.

Next, we identified relationships among microbes and metabolic pathways using multi-omics. *Bacteroidetes* were negatively correlated with phosphonate and phosphinate metabolism. *Verrucomicrobia* were positively correlated with the intestinal immune network for IgA production, fatty acid biosynthesis, and fatty acid elongation. *Firmicutes* were inversely correlated with phototransduction. *Cyanobacteria* were positively correlated with cysteine and methionine metabolism. *Tenericutes* were positively correlated with glycolysis/gluconeogenesis, fatty acid metabolism, terpenoid backbone biosynthesis, fructose and mannose metabolism, and purine metabolism and negatively correlated with primary bile acid biosynthesis. *Proteobacteria* were positively correlated with galactose metabolism, pentose and glucuronate interconversion, arginine biosynthesis, and primary bile acid biosynthesis. *Actinobacteria* were negatively correlated with vitamin B_6_ metabolism and glycolysis/gluconeogenesis and positively correlated with primary bile acid biosynthesis and drug metabolism (Fig. 5).

**FIG 6:**
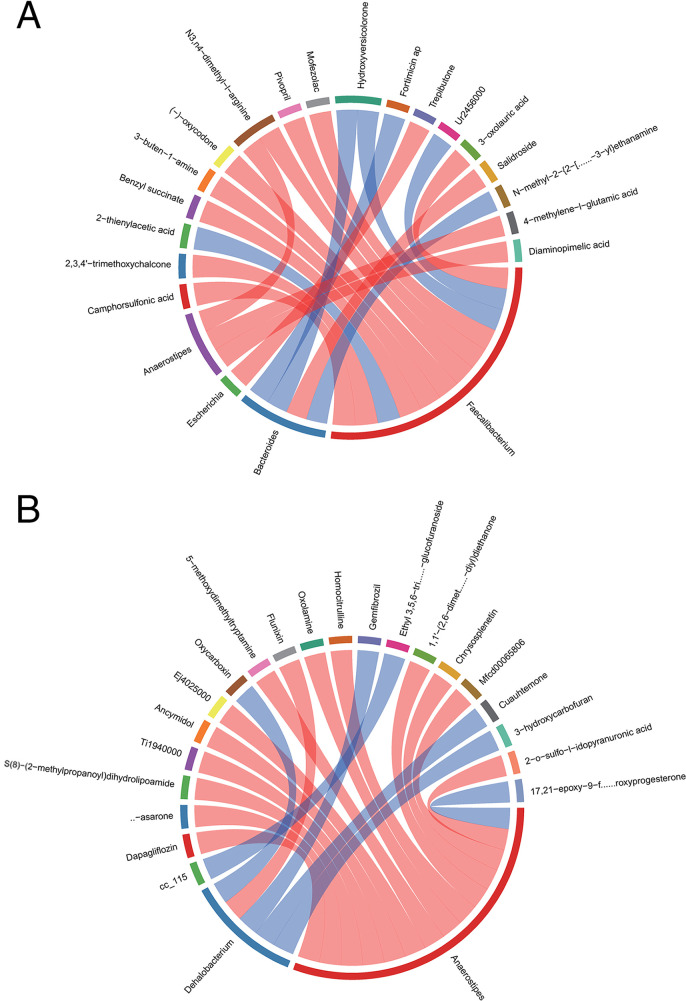

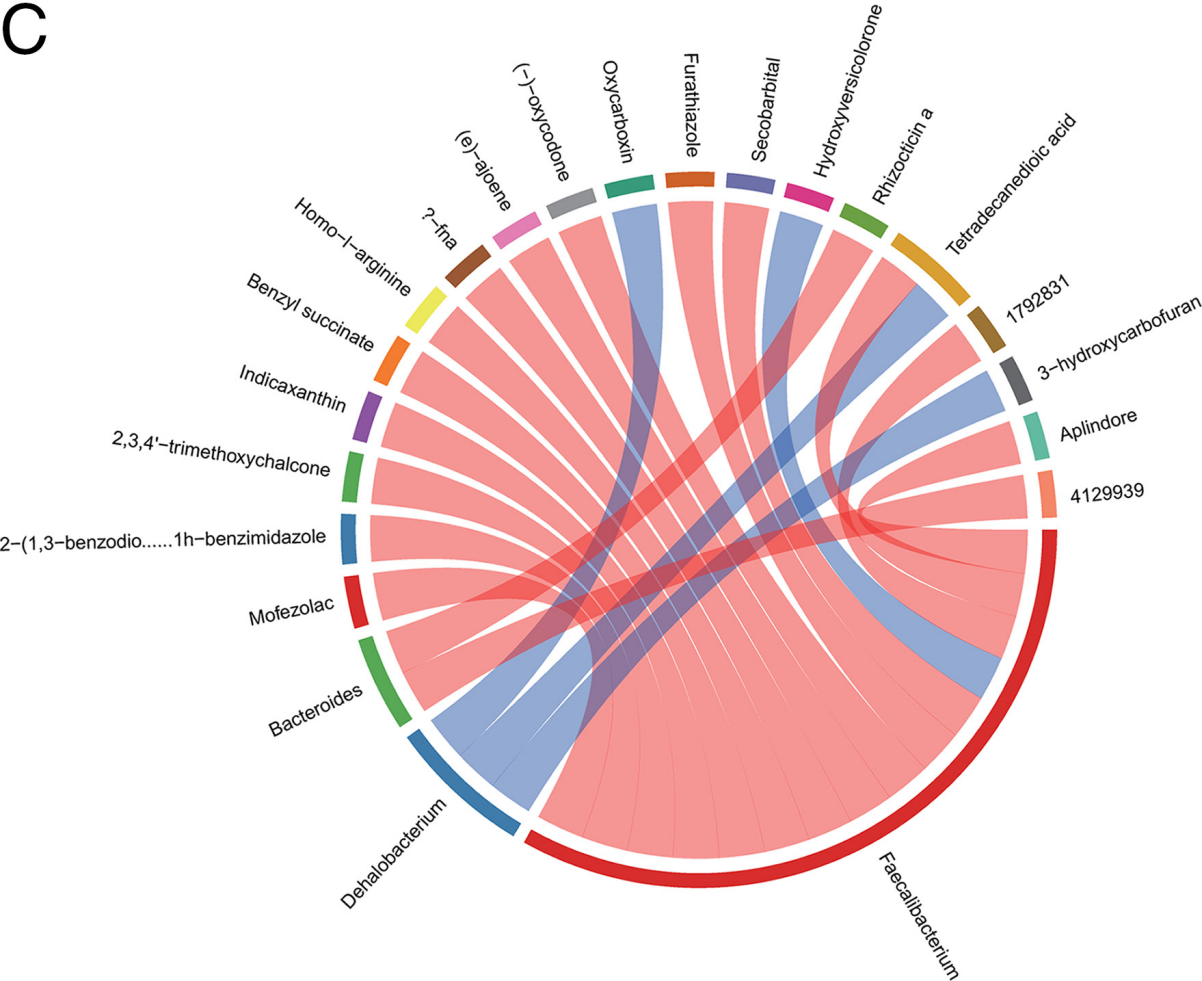
Rank correlation analysis was performed on differential metabolites and genus-level microbial groups. The top 20 relationship pairs with the strongest rank correlation (with the largest correlation coefficient absolute value and *P* < 0.05) are shown as chord diagrams. The blue line represents a positive correlation, and the red line represents a negative correlation. (A) Group E2 versus E1; (B) group E3 versus E1; (C) group E3 versus E2.

At the genus level, rank correlation analysis was performed on differential metabolites and microbial groups. In all three comparison groups (E2 versus E1, E3 versus E1, E3 versus E2), *Faecalibacterium*, *Bacteroides*, *Escherichia*, *Anaerostipes*, and *Dehalobacterium* were correlated with a variety of metabolites (Fig. 6).

## DISCUSSION

The gut microbiome plays vital roles in resistance to exogenous microorganisms via phage deployment, secretion of antibacterial substances, competing nutrients, and intestinal barrier function. Using antibiotics destroys the gut microbiome, making the conditions suitable for pathogenic microorganisms to colonize the intestine, and affects the metabolomics. It has been shown that differences in feed composition, broiler breed, age, and environment also alter chicken gut microbiota and metabolism (14, 15). However, their effects on gut microbiome and metabolome correlations need to be further investigated in the future. Here, we investigated the effects of enrofloxacin on the colonization of *S.* Typhimurium in the intestinal tracts of chickens. Our results showed that on day 7, *S.* Typhimurium was highly abundant in the cecum contents of chickens untreated with antibiotics. As the gut microbial community matured, the abundance of *S.* Typhimurium in untreated chickens was gradually decreased, whereas that in enrofloxacin-treated chickens gradually increased and persisted, similar to previous reports (6, 8, 16). Thus, our findings showed that different concentrations of enrofloxacin altered the ability of *S.* Typhimurium to colonize the gut in chickens. Enrofloxacin may cause severe *S.* Typhimurium infection.

On day 7, the highest diversity was observed in the high-dosage group, and the lowest diversity was observed in the low-dosage group. On day 21, the low-dosage group showed the highest diversity, whereas the high-dosage group showed the lowest diversity. These results are different from previous findings, potentially because of the use of antibiotics to challenge *Salmonella* in this study (6, 17, 18). Generally, the α-diversity of the cecum microbiota increases with age. A higher microbiota diversity is thought to be a marker of mature intestinal microbiota that is less sensitive to environmental factors and less susceptible to infection (19). Previous studies have shown that mice with lower intestinal microbiota complexity are less resistant to Streptococcus enterocolitis colonization and more susceptible to enterocolitis than normal mice (20). Thus, the use of enrofloxacin may have increased the risk of infection of chickens with pathogenic bacteria.

Our findings showed that using antibiotics significantly altered the microbial community structure, with clear differences according to the antibiotic concentration. The main reasons for the changes in microbial structure were decreased *Proteobacteria* and *Firmicutes* and increased *Bacteroidetes* at the three sampling points. Indeed, the early microbial community mainly comprised *Firmicutes* and *Proteobacteria*, accounting for more than 90% of the total sequence, whereas the late microbial community was dominated by *Bacteroidetes* and *Firmicutes*. Studies have shown that *Bacteroides* strains can produce propionate to reduce the colonization of *S.* Typhimurium (10). *Firmicutes* are primarily anaerobic bacteria. The anatomic physiology and feeding habits of chickens may explain the high abundance of *Firmicutes*. Turnbaugh et al. found that the appendixes of obese mice contain higher concentrations of acetic acid and butyric acid and that the ratio of *Firmicutes* to *Bacteroidetes* is increased in comparison with normal mice (21). Changes in the ratio of *Firmicutes* to *Bacteroidetes* in the experimental group may affect the accumulation of chicken fat and the production of SCFAs, which may explain the higher abundance of *S.* Typhimurium in group E2 on day 14. Additionally, on day 14, the abundances of *Enterobacteriaceae* in groups E2 and E3 were lower than that in group E1, although the difference was not significant. *Enterobacteriaceae* can inhibit the colonization of *Salmonella* in the intestine by competing for oxygen (13). Notably, the abundances of *Anaerotruncus*, *Butyricicoccus*, *Ruminococcus*, *Dorea*, and *Clostridium* were gradually decreased. In addition, higher concentrations of enrofloxacin resulted in lower abundances of *Ruminococcus* and *Anaerotruncus* on days 7 and 14, similar to a previous study of florfenicol (22).

Many anaerobic bacteria can produce SCFAs via microbial fermentation of carbohydrates, which have immunomodulatory and anti-inflammatory effects. Butyric acid can mediate the inhibition of *Salmonella* by downregulating the expression of pathogenicity island I and can also reduce *Salmonella*-induced proinflammatory responses to gut cells *in vitro* (23, 24). Some bacteria compete with *Salmonella* for oxygen and produce high concentrations of SCFAs to limit their colonization (25). SCFAs exert inflammatory effects by regulating the production of cytokines and prostaglandins (26) and can enhance host immune function. Sunkara et al. found that SCFAs reduce the *Salmonella* load in the gut contents and enhance the host’s defense ability against *Salmonella* (27). Butyrate can promote the β-oxidation of colonic epithelial cells, reduce the oxygen partial pressure in the gut cavity, and inhibit pathogenic bacteria (28). Any reduction in SCFA production could explain the high abundance of *Salmonella* in the cecum of antibiotic-treated chickens. In this study, on day 14, *Lactobacillus* and *Oscillospira* numbers were higher in groups E2 and E3 than in group E1, consistent with previous studies. This may be due to the microaerophilic growth of *Lactobacillus* and the production of reactive oxygen species by granulocytes in the infected chicken cecum to penetrate the site of inflammation and provide favorable growth conditions for lactic acid bacteria (25, 29, 30). Notably, accumulation of lactic acid may damage the gut defense barrier and increase the osmotic load in the gut cavity, and *Salmonella* can oxidize lactic acid to enhance its gut adaptability (7, 31). Thus, our results indicated that enrofloxacin decreased the colonization resistance of the original *S.* Typhimurium in the intestine, allowing *S.* Typhimurium to more easily colonize the intestine.

Metabolomic analysis showed that the differential metabolic pathways shared by groups E2 versus E1 and E3 versus E1 included linoleic acid metabolism and α-linolenic acid metabolism. According to several reports, polyunsaturated fatty acids, such as linoleic acid, α-linolenic acid, and γ-linoleic acid, are metabolized by macrophages into fatty acids with a ketone structure. Notably, fatty acids containing an enone structure have the most effective anti-inflammatory activity (32). Linolenic acid can kill Helicobacter pylori and reduce the bacterial load in the stomachs of mice. *In vivo*, treatment with linolenic acid reduces the levels of proinflammatory cytokines (33). Interestingly, addition of flaxseed oil to the diet of pigs recovered the levels of α-linolenic acid, eicosapentaenoic acid, and total n-3 polyunsaturated fatty acids in the gut tract and improved gut morphology. Flaxseed oil can also increase jejunal lactase activity and claudin-1 protein expression; downregulate the mRNA expression of gut necrosis signals; downregulate gut *TLR*4 mRNA, its downstream signals, and their adaptor molecules; and decrease the functionality of RIPK2 expression (34). Notably, the changes observed in the PPAR signaling pathway and amino acid biosynthesis were due to the metabolic effects of *S.* Typhimurium colonization in the chicken cecum. The abundance of α-linolenic acid was lower in groups E2 and E3 than in group E1 and lower in group E3 than in group E2. This decrease in the level of α-linolenic acid may be the reason for the high abundance of *S.* Typhimurium in groups E2 and E3. However, further studies are required to elucidate the effects of administering α-linolenic acid on *S.* Typhimurium colonization.

In this study, we found that IgA production by the *Verrucomicrobia* gut immune network was positively related to fatty acid biosynthesis. The phylum *Verrucomicrobia* contains only one member, i.e., Akkermansia muciniphila, which is a member of the human gut microbiota and a common bacterium that degrades mucins in the gut (35). It has been reported to be protective against diet-induced obesity (36, 37) and to promote mucosal wound healing (38) and antitumor responses during anti-programmed death-1 immunotherapy (39). Therefore, when enrofloxacin is administered, changes in *Verrucomicrobia* may affect *S.* Typhimurium colonization. Notably, *Faecalibacterium* was associated with the most metabolites in the E2 versus E1 and E3 versus E2 groups and *Anaerostipes* was associated with the most metabolites in the E3 versus E1 group. Several studies have reported that the metabolites secreted by Faecalibacterium prausnitzii attenuate inflammation in cells and 2,4,6-trinitrobenzenesulfonic acid-induced colitis models by blocking nuclear factor (NF)-κB activation and IL-8 production (40). Notably, *F. prausnitzii* secretes a protein with a molecular weight of 15 kDa, which mediates resistance to colitis in animal models by inhibiting the NF-κB pathway in gut epithelial cells (41). Moreover, *F. prausnitzii* can adapt to the mucus layer and ferment to produce butyrate. The absence of *F. prausnitzii* and other microorganisms with similar functions is often accompanied by inflammatory bowel disease and obesity (42). These bacteria may be the major bacteria affecting the gut metabolome following enrofloxacin administration.

Our results indicated that infection with *S.* Typhimurium after enrofloxacin administration affected the colony structure and metabolite composition in the chicken cecum, thereby altering *S.* Typhimurium colonization in the gut. The effects varied according to the concentration of enrofloxacin used. The colonies and metabolites identified in this study may provide important insights into the prevention and control of *S.* Typhimurium infection. The observed increase in *S.* Typhimurium infection induced by enrofloxacin also facilitates the more careful and rational use of antibiotics in poultry.

## MATERIALS AND METHODS

### Bacterial strain.

*S.* Typhimurium ATCC 14028 (stored in our laboratory) was incubated in lysogeny broth at 37°C for 16 h. For bacterial counting, the liquid was diluted and inoculated on XLT4 agar plates at 37°C for 24 h.

### Enrofloxacin exposure and infection in chickens.

**FIG 7:**
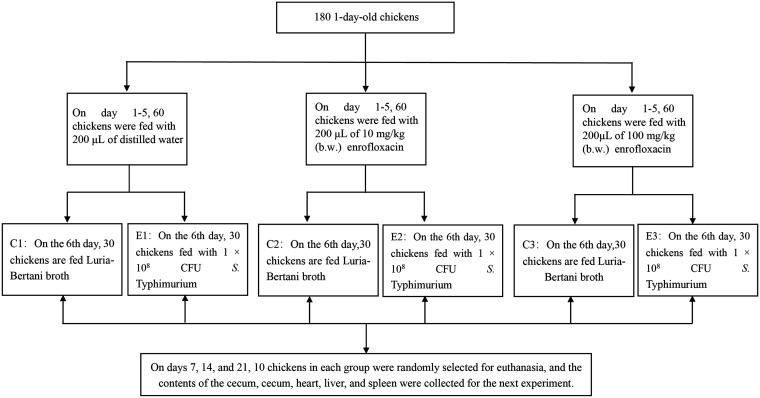
Animal experiment design. In total, 180 specific-pathogen-free chickens were randomly divided into six groups (C1, C2, C3, E1, E2, and E3). On days 1 to 5, the groups were fed with 200 μl of enrofloxacin or distilled water at different concentrations daily. On the 6th day, groups E1, E2, and E3 were fed 1 × 10^8^ CFU *S.* Typhimurium per chicken, and groups E1 and C1 were fed equal amounts of lactose broth. Samples were taken from each chicken at 7, 14, and 21 days of age.

The experimental protocol involving animals in this study was approved by the Animal Ethics Committee of Sichuan University. One-day-old chickens (*n* = 180) were randomly divided into six groups (*n* = 30 each). Each group was housed in a separate isolator containing drug-free feed and water. Each chicken was weighed, and the average body weight (b.w.) of the chickens was approximately 50 g. From 1 to 5 days of age, chickens were fed different concentrations for enrofloxacin (Baytril 10% injectable solution; Bayer AG, Leverkusen, Germany) (C1 and E1 [control], 200 μl distilled water; C2 and E2 [low dosage, 10 mg/kg b.w., 200 μl 2.5-mg/ml enrofloxacin]; C3 and E3 [high dosage, 100 mg/kg b.w., 200 μl 25-mg/ml enrofloxacin]) (43). At 6 days of age, chickens in groups E1, E2, and E3 were challenged with oral administration of 1 × 10^8^ CFU *S.* Typhimurium, whereas chickens in groups C1, C2, and C3 were given equal amounts of Luria-Bertani broth. All chickens were euthanized on day 7, 14, or 21 (*n* = 10/group at each time), and cecum contents, heart, spleen, liver, and ceca were collected. All cecum contents were collected from the same part of the cecum. The ileum and colon were rapidly clamped to avoid overflow of gastrogut digestive fluids, which can contaminate other parts of the intestine (Fig. 7).

### 16S rRNA amplicon sequencing.

Samples were collected from 10 chickens from the same group at each sampling time. According to the manufacturer’s instructions, a QIAamp DNA stool minikit (Qiagen, Hilden, Germany) was used to test 200-mg aliquots and 30 mg tissue from each chicken manure sample. Total genomic DNA was extracted from the samples, and the concentration and quality of the extracted DNA were measured using a NanoDrop spectrophotometer and agarose, followed by confirmation using gel electrophoresis. The extracted DNA was stored at −20°C until further analysis.

Using the isolated genomic DNA as the template, PCR amplification was performed on the V3-V4 hypervariable region of the bacterial 16S rRNA gene. The primers used were 338F (5′-ACTCCTACGGGAGGCAGCAG-3′) and 806R (5′-GGACTACHVGGGTWTCTAAT-3′). Sample-specific 7-bp barcodes were assigned to multiplex sequencing primers. The 25-μl PCR system and PCR parameters for PCR amplification were set up using Agencourt. AMPure XP magnetic beads were used for purification of PCR amplification products, and products were dissolved in elution buffer. The libraries were labeled, and the fragment range and concentration of the libraries were checked using an Agilent 2100 Bioanalyzer. The qualified libraries were sequenced on a HiSeq platform according to the inserted fragment size.

### Sequencing and bioinformatics analysis.

Raw reads were filtered to remove adaptors and low-quality and ambiguous bases, and then paired-end reads were added to tags by the Fast Length Adjustment of Short reads program (FLASH, v1.2.11) (44) to get the tags. The tags were clustered into OTUs with a cutoff value of 97% using UPARSE software (v7.0.1090) (45), and chimera sequences were compared with the Gold database using UCHIME (v4.2.40) (46) to detect. Then, OTU representative sequences were taxonomically classified using Ribosomal Database Project (RDP) Classifier v.2.2 with a minimum confidence threshold of 0.6 and trained on the Greengenes database v201305 by QIIME v1.8.0 (47).The USEARCH_global (48) was used to compare all tags back to OTU to get the OTU abundance statistics table of each sample. α- and β-diversity were estimated by MOTHUR (v1.31.2) (49) and QIIME (v1.8.0) (47) at the OTU level, respectively. Sample clustering was conducted by QIIME (v1.8.0) (47) based on unweighted pair group method using average linkages (UPGMA). The barplot and heatmap of different classification levels were plotted with R package v3.4.1 and R package “gplots,” respectively. LEfSe cluster analysis or LDA was conducted by LEfSe.

### Untargeted metabolomics for chicken cecal content.

After thawing samples slowly at 4°C, 25 mg was measured and added to a 1.5-ml Eppendorf tube. Then, 800 μl extract (methanol-acetonitrile/-water, 2:2:1, vol/vol/vol, precooled at −20°C), 10 μl internal standard 1, and 10 μl internal standard 2 were added. Two small steel balls were then added, and samples were ground in a tissue grinder (50 Hz, 5 min), sonicated at 4°C for 10 min, and placed in a freezer at −20°C for 1 h. After centrifugation, 600 μl supernatant was removed, placed in a refrigerated vacuum concentrator, and dried. Next, 200 μl of a double solution (methanol-H_2_O, 1:9, vol/vol) was added for recombination, and the samples were vortexed for 1 min and sonicated for 10 min at 4°C. The supernatants were then centrifuged at 25,000 × *g* for 15 min at 4°C and placed in a sample vial. Then, 20 μl supernatant from each sample was mixed with quality control samples to assess the repeatability and stability of the liquid chromatography-mass spectrometry (LC-MS) analysis. A Waters two-dimensional (2D) ultraperformance liquid chromatograph (UPLC) (Waters, USA) in tandem with a Q Exactive Plus high-resolution mass spectrometer (Thermo Fisher Scientific, USA) was used for the separation and detection of metabolites. All LC-MS/MS mass spectrometry raw data (original files) were sent to Compound Discoverer (v.3.0; Thermo Fisher Scientific) for data processing. Identification of metabolites was performed using several databases. Results from Compound Discoverer v.3.0 were imported into metaX (50) for data preprocessing. All the identified metabolites were classified and annotated with reference to the Kyoto Encyclopedia of Genes and Genomes (KEGG) and HMDB databases to understand the classification of the metabolites. Multivariate statistical analysis (principal-component analysis [PCA] and partial least-squares discriminant analysis [PLS-DA]) (51, 52) and univariate analysis (fold change [FC] and Student’s *t* tests) were used to screen for between-group differential metabolites. Data were analyzed using clustering analysis, log_2_ conversion, and *z*-score normalization (zero mean normalization). Hierarchical clustering was used in the clustering algorithm, and the Euclidean distance was used to calculate the metabolic pathway enrichment of differential metabolites in the KEGG database. Metabolic pathway enrichment analysis of differential metabolites was based on the KEGG database, and metabolic pathways with *P < *0.05 were those significantly enriched for differential metabolites.

### Bacterial identification and enumeration.

First, 200 mg organ tissue and cecum content from each animal was added to 10 ml sterile saline and shaken well. Then, 1 ml bacterial solution was added to 9 ml sterile saline for homogenization; samples were serially diluted and spread on XLT4 agar plates. The medium was then incubated for 24 h under aerobic conditions at 37°C, and *S.* Typhimurium colonies were counted. The results are expressed as log_10_ CFU/g. All analyses were performed in triplicate.

### Correlation analysis between the microbiome and metabolome.

The metabolites were pretreated with positive and negative ions collected from the metabolome to obtain metabolites. The metabolites identified in the metabolome were down-treated using gene coexpression network analysis to obtain coexpression clusters. Metabolites were annotated to the metabolic pathway, and pathway abundance was calculated based on metabolite intensity. Correlations between metabolite intensity and microbial abundance were assessed using Spearman’s correlation analysis.

### Statistical analysis.

*S.* Typhimurium count results are expressed in log_10_ CFU/g. One-factor analysis of variance (ANOVA) was performed on the above results using SPASS 20.0.
