# Supplementary material for: Enrofloxacin Shifts Intestinal Microbiota and Metabolic Profiling and Hinders Recovery from Salmonella enterica subsp. enterica Serovar Typhimurium Infection in Neonatal Chickens
Source: mSphere. 2020 Sep 9;5(5):e00725-20. doi: 10.1128/mSphere.00725-20 (PMC7485687; doi:10.1128/mSphere.00725-20)
Supplement: TABLE S1 [file mSphere.00725-20-st001.pdf]

Table S1

Taxa	Groups								
**Phylum**	**7E1**	**7E2**	**7E3**	**14E1**	**14E2**	**14E3**	**21E1**	**21E2**	**21E3**
Verrucomicrobia	0	0	0	4.94E-06	2.16E-05	2.53E-06	0	0	7.31E-06
Thermi	4.88E-06	1.97E-05	4.32E-05	0	0	0	0	0	0
Actinobacteria	2.20E-05	2.47E-06	4.56E-05	0.000699	0.001409	0.000169	0.000206	0.000189	0.000193
Tenericutes	0	0	0	0.000215	0.002341	0.002508	0.001622	0.007415	0.003503
Cyanobacteria	9.76E-06	7.40E-06	9.61E-06	2.47E-05	0.001692	0.003699	0.000861	0.012317	0.010128
Proteobacteria	0.073029	0.065214	0.063566	0.121165	0.013446	0.027343	0.002524	0.011835	0.012907
Bacteroidetes	0.00021	5.67E-05	0.000142	3.70E-05	0.295526	0.017371	0.412781	0.364313	0.420906
Firmicutes	0.926724	0.9347	0.936194	0.877855	0.685564	0.948906	0.582005	0.60393	0.552356
