# Supplementary material for: Enrofloxacin Shifts Intestinal Microbiota and Metabolic Profiling and Hinders Recovery from Salmonella enterica subsp. enterica Serovar Typhimurium Infection in Neonatal Chickens
Source: mSphere. 2020 Sep 9;5(5):e00725-20. doi: 10.1128/mSphere.00725-20 (PMC7485687; doi:10.1128/mSphere.00725-20)
Supplement: TABLE S2 [file mSphere.00725-20-st002.pdf]

Table S2

Taxa	Groups								
**Genus**	**7E1**	**7E2**	**7E3**	**14E1**	**14E2**	**14E3**	**21E1**	**21E2**	**21E3**
Blautia	0.0086	0.008677	0.007694	0.008083	0.005315	0.004526	0.003507	0.01096	0.003232
Anaerotruncus	0.053596	0.006568	0.005869	0.005035	0.00498	0.009032	0.003572	0.002186	0.001475
Butyricicoccus	0.018525	0.039124	0.031825	0.012376	0.009924	0.027556	0.004913	0.002793	0.00282
Coprococcus	0.00061	0.005608	0.014108	0.009404	0.009873	0.007727	0.016694	0.025795	0.013953
Dorea	0.02435	0.027667	0.014975	0.013642	0.011429	0.01704	0.004239	0.01293	0.006321
Clostridium	0.039958	0.066906	0.071992	0.030218	0.020434	0.026701	0.00648	0.011062	0.005355
Escherichia	0.044337	0.043481	0.04594	0.120792	0.013298	0.02706	0.002471	0.01167	0.012849
Lactobacillus	0.008297	0.012528	0.008847	0.008733	0.044324	0.11483	0.197679	0.131528	0.26228
Oscillospira	0.23907	0.215051	0.171717	0.070295	0.034772	0.08137	0.043218	0.048907	0.032091
Ruminococcus	0.216861	0.164213	0.180617	0.159861	0.128676	0.109191	0.083451	0.07457	0.037337
Bacteroides	0.00021	5.67E-05	0.000142	2.72E-05	0.29548	0.017368	0.412781	0.364296	0.420901
Other	0.345586	0.410121	0.446275	0.561534	0.421496	0.557598	0.220997	0.303303	0.201387
