# Supplementary figures and images for: Enrofloxacin Shifts Intestinal Microbiota and Metabolic Profiling and Hinders Recovery from Salmonella enterica subsp. enterica Serovar Typhimurium Infection in Neonatal Chickens
Source: mSphere. 2020 Sep 9;5(5):e00725-20. doi: 10.1128/mSphere.00725-20 (PMC7485687; doi:10.1128/mSphere.00725-20)

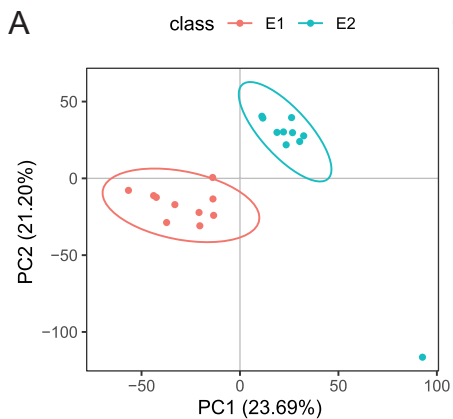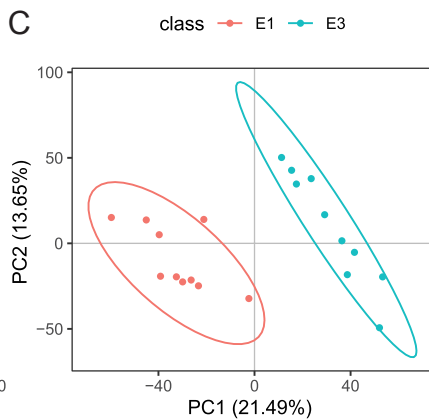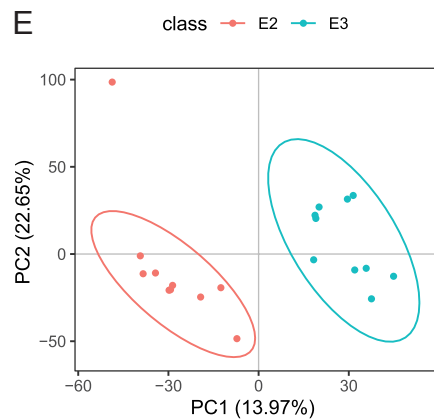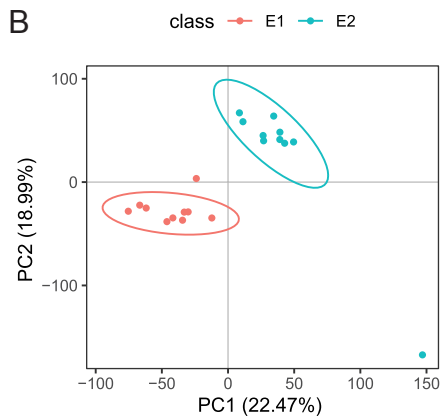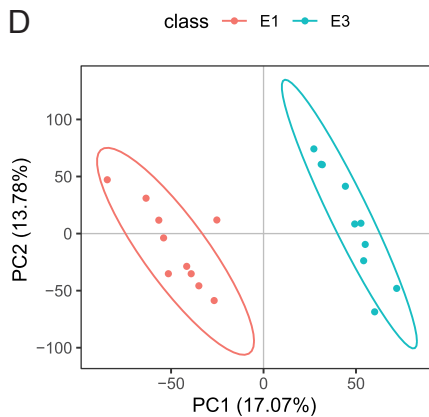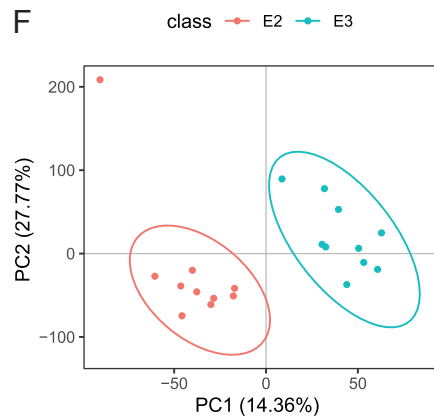

Supplement: FIG S1 [file mSphere.00725-20-sf001.pdf]
